# Cardiovascular outcomes in long COVID-19: a systematic review and meta-analysis

**DOI:** 10.3389/fcvm.2025.1450470

**Published:** 2025-01-29

**Authors:** Ting Zhang, Zhimao Li, Qimin Mei, Joseph Harold Walline, Zhaocai Zhang, Yecheng Liu, Huadong Zhu, Bin Du

**Affiliations:** ^1^ State Key Laboratory of Complex Severe and Rare Diseases, Emergency Department, Peking Union Medical College Hospital, Chinese Academy of Medical Science and Peking Union Medical College, Beijing, China; ^2^ Department of Emergency Medicine, Penn State Health Milton S. Hershey Medical Center and Penn State College of Medicine, Hershey, PA, United States; ^3^ Department of Critical Care Medicine, Second Affiliated Hospital, Zhejiang University School of Medicine, Hangzhou, Zhejiang, China

**Keywords:** COVID-19, cardiovascular, long-COVID, meta-analysis, COVID-19 management

## Abstract

**Introduction:**

There is growing evidence that patients with SARS-CoV-2 (The severe acute respiratory syndrome coronavirus 2) may have a variety of cardiovascular complications in the post-acute phase of COVID-19, but these manifestations have not yet been comprehensively characterized.

**Methods:**

We performed a systematic review and meta-analysis of primary research papers which evaluated individuals at least four weeks after confirmed COVID-19 diagnosis and reported on cardiovascular disease prevalence. Systematic search conducted without language restrictions from December 1, 2019 to June 31, 2022 on PubMed, EMBASE, Web of Science, Cochrane library, ProQuest Coronavirus Research Database, COVID-19 Living Overview of the Evidence (L-OVE) subset of Episteminokos and the World Health Organization (WHO) Covid-19 databases. Study was reported according to MOOSE-lists and the PRISMA guidelines. The risk of bias was identified using the Newcastle-Ottawa Scale (NOS) for observational studies. Random-effects meta-analyses examined the pooled risk difference in the prevalence of each symptom or symptom combination in cases with confirmed SARS-coV-2 infection compared with controls.

**Results:**

Eight cohort studies were eligible, including nearly 10 million people. Long COVID-19 was associated with a higher risk of thromboembolic disorders [HR 3.12 (1.60, 6.08)], coronary heart disease [HR 1.61 (1.13, 2.31)], stroke [HR 1.71 (1.07,2.72)], arrhythmia [HR 1.60 (1.13, 2.26)], cardiomyopathy [HR 1.71 (1.12, 2.61)], myocarditis [HR 6.11 (4.17,8.94)], hypertension [HR 1.70 (1.56, 1.85)], heart failure [HR 1.72 (1.15,2.59)] and cardiogenic shock [HR 2.09 (1.53,2.86)] compared to non-COVID-19 controls. Pooled risk differences in long COVID cases compared to controls were significantly higher for cardiomyopathy [0.15% (0.06, 0.23)], deep vein thrombosis [0.45% (0.06, 0.83)] and hypertension (0.32%, (0.06, 0.58) but not for thromboembolic disorders, coronary disease, stroke, arrhythmia, cardiomyopathy, myocarditis, hypertension, heart failure or cardiogenic shock.

**Conclusion:**

The risk of cardiovascular disease increased significantly four weeks or more after recovering from acute COVID-19. Care for survivors after an acute attack of COVID-19 should include paying close attention to cardiovascular health and disease.

**Systematic Review Registration:**

PROSPERO [CRD42022353965].

## Introduction

Worldwide, there have been hundreds of millions of people infected with COVID-19, and some people have reported an incomplete recovery even a few months after acute illness, a condition referred to as “long COVID-19” (1–4). Emerging data suggests that the acute sequelae of COVID-19 can involve the lungs and multiple extrapulmonary organs, including the cardiovascular system (5–8). The long-term effects of COVID-19 on cardiovascular diseases are becoming a major issue of global concern (5, 7–9). The available evidence shows that COVID-19 has adverse effects on the cardiovascular system, and confirms that the incidence of thromboembolic complications, acute coronary syndrome and myocarditis in the course of SARS-CoV-2 infection is increased, and that the prognosis for those with such complications and acute disease is poor (10). However, the full extent of cardiovascular involvement in long COVID-19 has not been determined. Although some studies have reported cardiovascular outcomes with long COVID-19, there is considerable heterogeneity among studies in terms of follow-up duration and population selection.

At this juncture, approximately 2 years into the COVID-19 pandemic, numerous large, high-quality studies on cardiovascular diseases in long COVID-19, with substantial follow-up time, have been conducted and published. A standardized case definition for the syndrome has not yet been universally accepted, leading to a lack of consensus. However, various classifications are emerging (11). In the United Kingdom, the National Institute for Health and Care Excellence (NICE) working guidelines have introduced specific terminology to describe post COVID-19 syndrome. They define “Ongoing symptomatic COVID-19” as the persistence of signs and symptoms for a duration of 4–12 weeks from the onset of infection. Additionally, “Post COVID-19 syndrome” is defined as signs and symptoms that continue beyond 12 weeks from the initial onset (11). Conversely, the US Centers for Disease Control and Prevention (CDC) utilize the term “Post COVID-19 Conditions” as an inclusive phrase encompassing a broad spectrum of health consequences that manifest more than four weeks after acute infection (11). Herein, we will refer to long COVID-19 as present in those “the continuation or development of new symptoms 3 months after the initial SARS-CoV-2 infection, with these symptoms lasting for at least 2 months with no other explanation” (12, 13). This systematic review and meta-analysis study aimed to study the risk of cardiovascular outcomes in long COVID-19.

## Methods

This systematic review was reported according to MOOSE-lists (14) and the PRISMA guidelines (15). The study protocol was registered with PROSPERO on August 1, 2022 (Reference: CRD42022353965).

### Eligibility

Inclusion criteria included studies that met the following criteria:.

1. Population: age ≥18 years old with confirmed evidence of SARS-CoV-2 infection [positive reverse transcription polymerase chain reaction (RT-PCR) or serology test] or probable COVID-19 (clinician defined or suspected COVID-19) who have persistent symptoms as defined by the study authors.
2. Study type: Prospective or retrospective cohort studies.
3. The follow-up time was at least 4 weeks (28 days) after the index date.
4. Outcomes: Cardiovascular diseases (thromboembolic disorders, coronary disease, stroke, arrhythmia, hypertension, heart failure, cardiogenic shock, cardiomyopathy and myocarditis) described in each eligible study.

Exclusion criteria for COVID-19:

1. Incomplete or inaccurate quantitative data (i.e., no exact proportions provided).
2. Outcomes precede exposure (i.e., cardiovascular outcomes were present prior to COVID-19 infection).
3. COVID-19 not verified by laboratory testing or ICD-10 linkage or was not clinically diagnosed.
4. No prior history of cardiovascular events.

### Search strategy

A systematic search was conducted by the primary reviewer (TZ) from December 1, 2019 to July 31, 2022 in seven electronic databases: PubMed, EMBASE, Web of Science, Cochrane Covid-19 Study Registry, ProQuest Coronavirus Research Database, COVID-19 Living Overview of the Evidence (L-OVE) subset of Episteminokos and the World Health Organization (WHO) Covid-19: Global literature on coronavirus disease. The search string implemented was: “long covid” OR “persistent covid” OR “post covid” OR “post-acute sequelae of SARS-CoV-2 PASC” OR “enduring COVID-19 sequelae” OR “long-haul covid” OR “long-tail covid”. No language or publication date restrictions were imposed (see Supplementary Material for complete search wording).

### Study selection and data extraction

Titles and abstracts of all studies were screened independently by TZ and independently verified by a second reviewer (ZML), with disagreements resolved by consensus or a third reviewer (YCL). Data including methods of diagnosis of infection, recruitment source, study characteristics, symptom prevalence and population demographics, were extracted independently by TZ and ZML with disagreements resolved by consensus.

### Risk of bias

The methodological quality of included studies was assessed independently by TZ and a second assessor (ZML) using the Newcastle-Ottawa Scale (NOS) for observational studies, with discrepancies resolved through discussimon (16, 17).

### Data synthesis and analysis

Meta-analysis was conducted if at least two articles were investigating the same symptom. Participants with confirmed SARS-CoV-2 infection (cases) were compared with subjects who tested negative for SARS-CoV-2 (controls). The DerSimonian–Laird (18) random effects model was used to pool hazard ratios (HRs) on the log scale (18). Risk ratios were taken as hazard ratios assuming no censoring (19). We also used random-effects meta-analyses to examine the pooled risk difference in the prevalence of each symptom or symptom combination in cases with confirmed SARS-coV-2 infection compared with controls. I^2^ estimates the proportion of the variance across study estimates that is due to heterogeneity and was considered as small if *I*^2^ < 50%, and large if statistical heterogeneity between the results of the studies was *I*^2^ ≥ 50%. We did not investigate publication bias given the recency of this literature and due to poor performance of standard tests for publication bias in prevalence studies (20).

All risk estimates were calculated with the corresponding 95% confidence intervals (CIs) and 95% prediction intervals (PIs). *P*-values <0.05 were considered statistically significant. All statistical analyses were performed with R software (version 4.1.2, Vienna, Austria) (21, 22), using the packages “metafor”, “metaviz” and “metaUtility”.

### Patient and public involvement

Patients were not involved in the design and conduction of this study.

## Results

**Figure 1:**
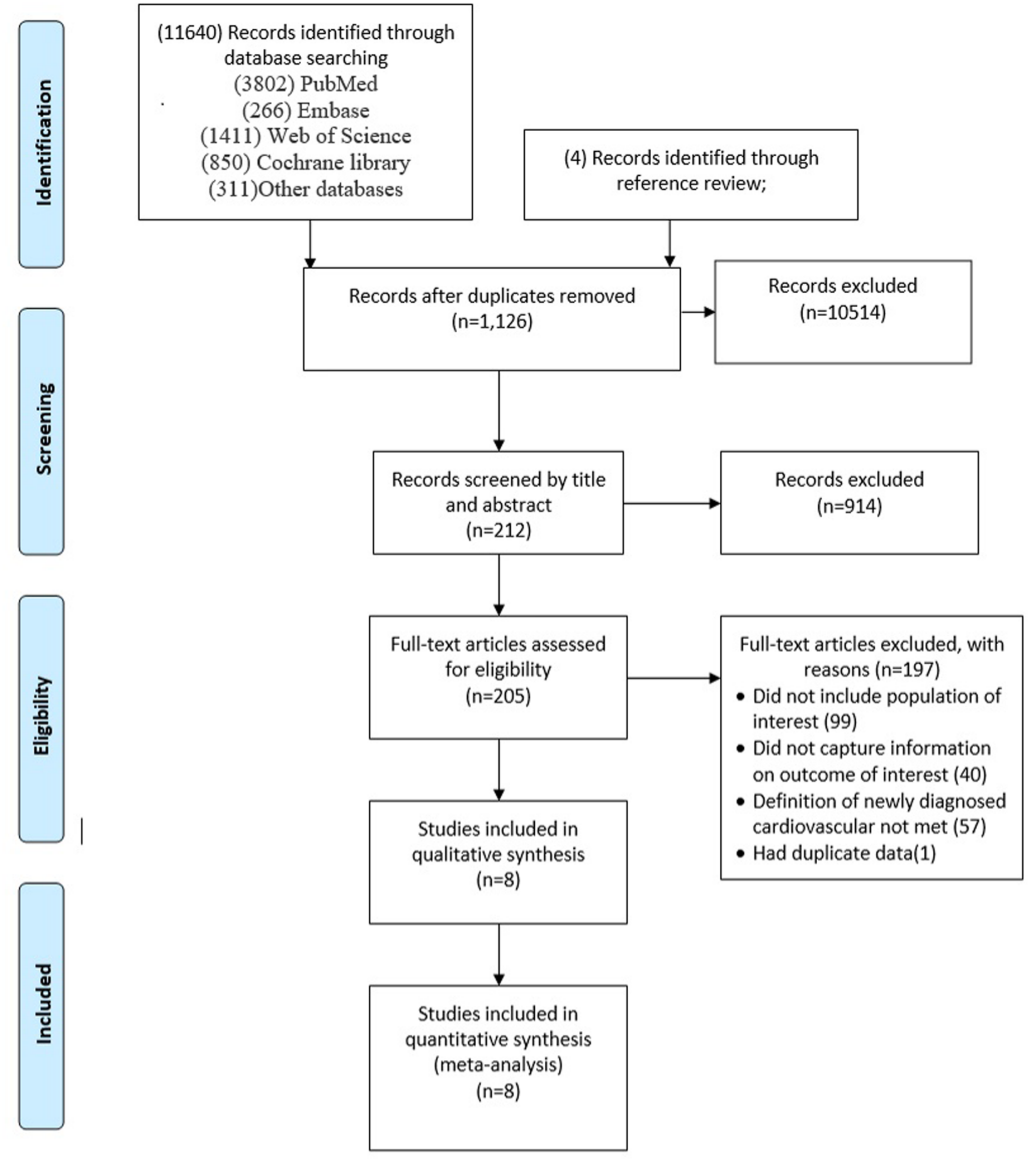
Study and participant selection.

The search yielded 11,640 citations. After duplicates were removed and titles and abstracts were reviewed, 11,428 articles were excluded. Of the remaining 212 studies, full-text articles for all 205 were available. Of these, 197 were then excluded after reviewing the full-text manuscripts. After several stages of review, eight (23–30) eligible studies were included in the meta-analysis (Figure 1; Table 1). This meta-analysis incorporates data from eight studies that utilized a control group design. The analysis encompasses a vast participant pool of 10 million individuals aged between 35 and 75 years. The follow-up period for the included studies ranges from 2 to 12 months.

**Table 1:** Characteristics of total study population.

First author (year)	Group	Population	Age (years), mean (SD or range) or median (IQR)	Gender (proportion)	Follow-up time	Study design
Xie et al. 2022 (30)	COVID-19 group	153,760	61.42 (15.64)	Male: 136,912 (89.04), Female: 16,848 (10.96)	347 (IQR 317–440) days	Cohort study
	Control group	5,637,647	63.46 (16.23)	Male: 5,091,519 (90.31), Female: 546,128 (9.69)	348 (318–441) days	
Rezel-Potts et al. 2022 (29)	COVID-19 group	428,650	35 (22–50)	Male: 217,782 (44.8), Female: 268,367 (55.2)	119 (IQR 0–210) days	Cohort study
	Control group	428,650	35 (22–50)	Male: 868,617 (44. 7), Female: 1,075,963 (55.3)	161 (IQR 4–225) days	
Subramanian, 2022 (26)	COVID-19 group	486,149	Not reported	Male:1,081,608 (46.1); Female:12,64,069 (53.9)	0.29 years (IQR 0.24–0.42)	Cohort study
	Control group	1,944,580	Not reported	Male:10,579,475 (45.9); Female:12,491,493 (54.1)	0.29 years (IQR 0.24–0.41)	
Walker, 2021	COVID-19 group	31,716	Not reported	Male:15,016 (47.3); Female:16,700 (52.7)	Defined by the study authors	Cohort study
	Control group	158,551	Not reported	Male:75,062 (47.3); Female:83,489 (52.7)	Defined by the study authors	
Cohen et al. 2022 (25)	COVID-19 group	87,337	75 (71–82)	Male: 58,110 (44)	64 (IQR 23–150) days	Cohort study
	Control group	87,337	74 (70–80)	Male: 1,169,435 (42)	64 (IQR 23–150) days	
Rivera et al. 2022 (27)	COVID-19 group	453	61.2 (14.3)	Male: 260 (57.4); Female: 193 (42.6)	12 months	Cohort study
	Control group	453	55.9 (17.8)	Male: 211 (46.6); Female: 242 (53.2)	12 months	
Ayoubkhani et al. 2021 (23)	COVID-19 group	36,100	60.9 ± 17.02	Males (54.9)	140 days	Cohort study
	Control group	36,100	61.5 ± 17.08	Males (54.9)	140 days	
Dautherty 2021 (28)	COVID-19 group	193,113	41.7 ± 13.9	Males (47.6)	87 (IQR 45–124) days	Cohort study
	Control group	193,113	41.6 ± 13.8	Males (47.5)	87 (IQR 45–124) days	

The risk of bias in included cohort studies assessed using the Newcastle–Ottawa Scale is presented in Supplementary Table S1. The overall score was 70 of 72 (97.2%), which is a low risk for bias.

### Thromboembolic disorders

**Figure 2:**
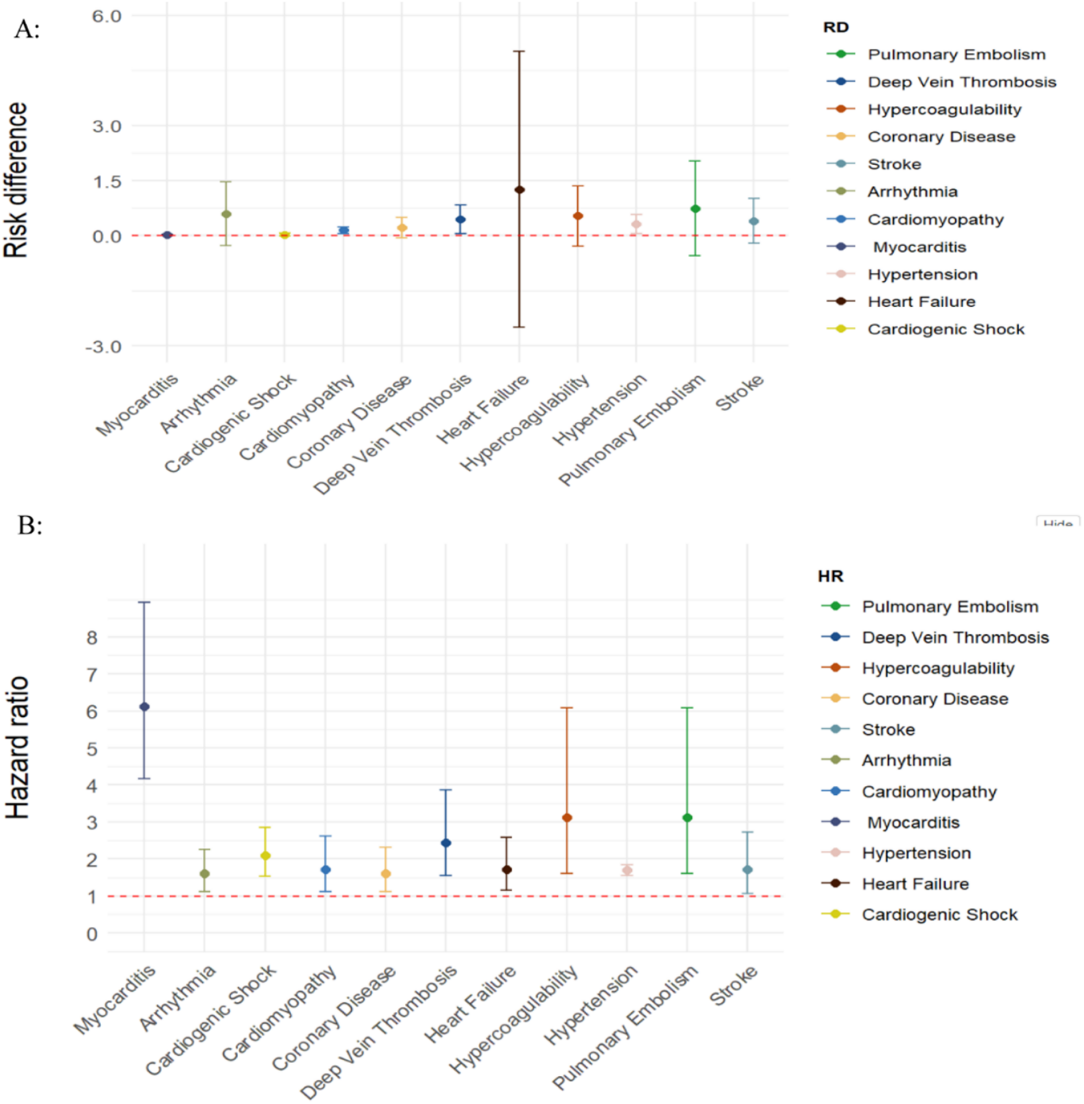
SARS-CoV-2 group vs. comparison group for risk difference per 100 individuals **(A)** and hazard ratio **(B)** for clinical sequelae in long COVID-19.

**Figure 3:**
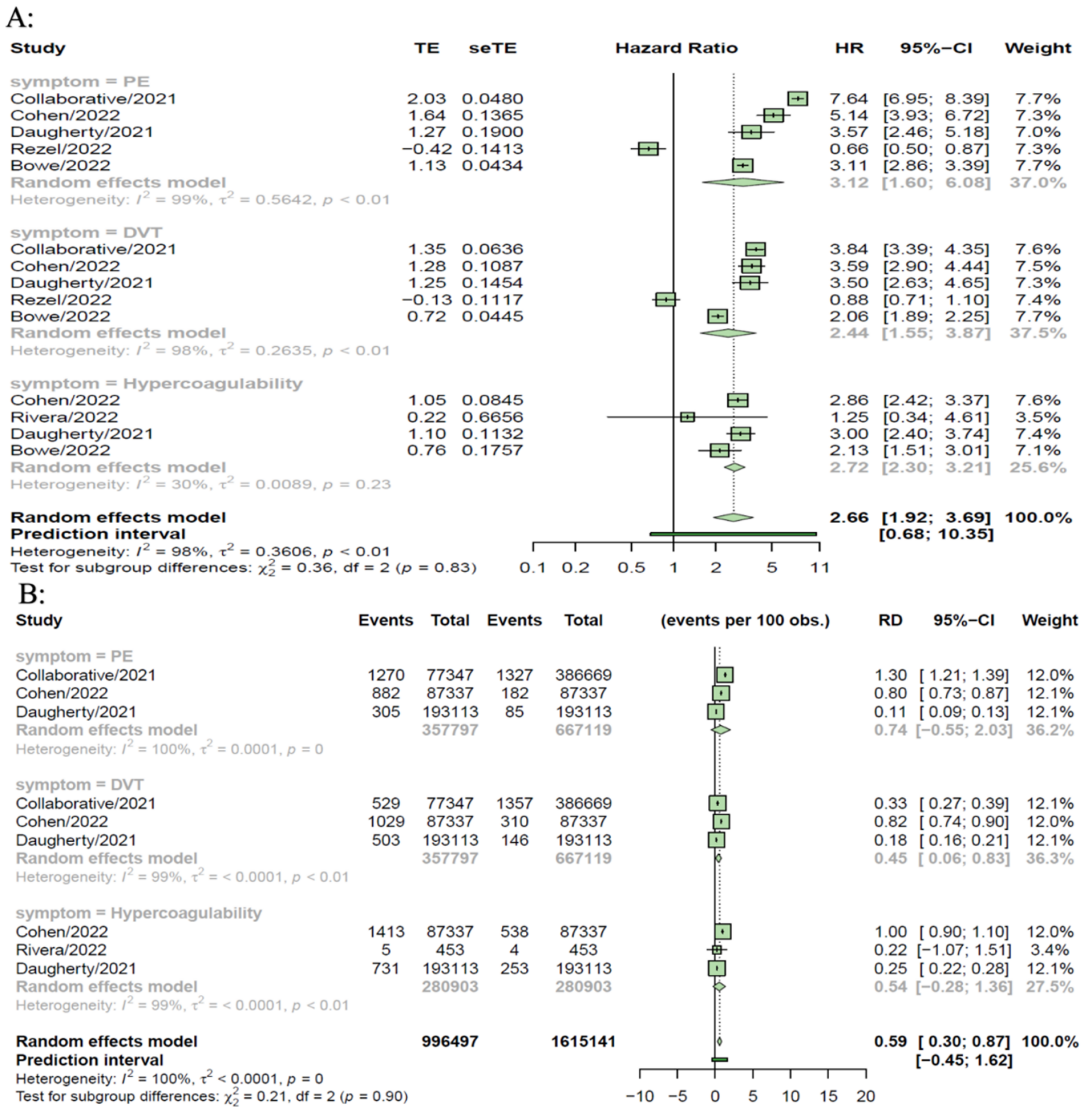
SARS-CoV-2 group vs. comparison group for risk difference per 100 individuals **(A)** and hazard ratio **(B**) for thromboembolic disorders in long COVID-19.

Six controlled studies provided data on thromboembolic disorders. Thromboembolic disorders included pulmonary embolism [HR = 3.12 (1.60, 6.08), RD 0.74% (−0.55, 2.03)]; deep vein thrombosis [HR = 2.44 (1.55, 3.87), RD = 0.45% (0.06, 0.83)] and hypercoagulability [HR = 2.72 (2.30, 3.21); RD = 0.54% (−0.28, 1.36)]. The risks and burdens of a composite of these thromboembolic disorders were 3.12 (1.60, 6.08) and 0.59% (0.30, 0.87) (see Figures 2, 3).

### Coronary disease

Five controlled studies provided data on coronary disease. In long COVID-19, the pooled risk of coronary heart disease was 1.61 (1.13, 2.31) times non-COVID-19 patients and the risk difference between them was 0.22% [95%CI (−0.06, 0.50)] (see Figure 2; Supplementary Figure S1).

### Stroke

Five controlled studies provided data on stroke. In long COVID-19, the pooled risk of stroke was 1.71 (1.07, 2.72) times higher than that of non-COVID-19 patients and the risk difference was 0.40% [95%CI (−0.21, 1.01)] (see Figure 2; Supplementary Figure S2).

### Arrhythmia

Five controlled studies provided data on arrhythmia. In long COVID-19, the pooled risk of arrhythmia was 1.60 (1.13, 2.26) times higher than that of non-COVID-19 patients, and the risk difference was 0.59% (95%CI (−0.27, 1.46) (see Figure 2; Supplementary Figure S3).

### Cardiomyopathy and myocarditis

Four controlled studies provided data on cardiomyopathy and myocarditis. In long COVID-19, the pooled risk of cardiomyopathy was 1.71 (1.12, 2.61) times higher than that of non-COVID-19 patients and the risk difference between them was 0.15% (0.06, 0.23) (see Figure 2). Three controlled studies provided data on myocarditis. The pooled risk of myocarditis was 6.11(4.17, 8.94) times higher than that of non-COVID-19 patients and the risk difference between them was 0.03% (0.02, 0.04) (see Figure 2; Supplementary Figure S4).

### Hypertension

Only two controlled studies provided data on hypertension. In long COVID-19, the pooled risk of hypertension was 1.70 times higher than that of non-COVID-19 patients (1.56, 1.85), and the risk difference between them was 0.32% (0.06,0.58) (see Figure 2; Supplementary Figure S5).

### Heart failure

Five controlled studies provided data on heart failure. In long COVID-19, the pooled risk of heart failure was 1.72 (1.15, 2.59) times higher than that of non-COVID-19 patients and the risk difference between them was 1.26% (−2.50, 5.03) (see Figure 2; Supplementary Figure S6).

### Cardiogenic shock

Three controlled studies provided data on cardiogenic shock. In long COVID-19, the pooled risk of cardiogenic shock was 2.09 (1.53, 2.86) times higher than that of non-COVID-19 patients, and the risk difference between them was 0.03% (−0.01, 0.07) (see Figure 2; Supplementary Figure S7).

## Discussion

In this meta-analysis of studies examining patients four weeks or more after COVID-19 infection, we found an increased risk of new-onset cardiovascular diseases, including stroke, arrhythmia, myocarditis, cardiomyopathy, coronary heart disease, hypertension, heart failure, thromboembolic disease, and cardiogenic shock. The current pooled results showed that compared with non-COVID-19 controls, the incidence of cardiovascular diseases in long COVID-19 is increased, except for myocarditis, hypertension, DVT.

Evidence from prior studies has demonstrated an association between individuals with cardiovascular disease and an unfavorable prognosis amid acute COVID-19 infection (31–33). Our meta-analysis shows that the risk of cardiovascular disease goes well beyond the acute phase of COVID-19. First, our results emphasize the need to continue to optimize primary prevention strategies for SARS-CoV-2 infection; that is, the best way to prevent long COVID and its numerous complications, including a higher risk of cardiovascular sequelae, is to prevent SARS-CoV-2 infection in the first place (34). Second, the hazard risk of sequelae of these events, such as hypertension, stroke and thromboembolic disease, was almost 2–3 times higher than in a non-COVID-19 population. Given the large and increasing number of COVID-19 patients, these findings suggest that more and increasing medical resources may be needed to deal with cardiovascular complications in COVID-19 survivors.

The mechanism of the association between COVID-19 and the development of cardiovascular disease after the acute phase of the disease is not fully understood. Oikonomou et al. (7) conducted a prospective study to evaluate vascular endothelial dysfunction in COVID-19 patients during long-term follow-up after SARS-COV-2 infection. Common laboratory markers of COVID-19 severity, as well as specific endothelial and inflammatory biomarkers, were also experimentally evaluated. These studies found that endothelial function was impaired in patients with SARS-CoV-2 by brachial artery flow-mediated dilation (FMD) assessment compared with non-COVID-19 controls matched by propensity scores, accompanied by a high level of inflammation that may lead to coagulation dysfunction and micro-thrombosis (10, 35). In the multicenter autopsy study conducted by Basso et al., 14% of cases had myocarditis (defined as lymphocyte infiltration and cardiomyocyte necrosis), 86% had interstitial macrophage infiltration, and 19% had pericarditis and right ventricular injury (36). At present, the speculated mechanisms of cardiovascular complications include direct viral invasion of cardiomyocytes and subsequent cell death, endothelial cell infection and endothelin/complement activation and complement-mediated coagulation and microvascular disease, elevated levels of proinflammatory cytokines and activation of TGF-β signal transduction through the Smad-signaling pathway, resulting in subsequent cardiac fibrosis and scar formation (36). These mechanisms of action may result in abnormally persistent hyperactivated immune responses, autoimmunity, or the persistence of the virus at immunologically privileged sites (37–43). These mechanisms can explain the scope of long COVID-19 cardiovascular sequelae investigated in this meta-analysis (44). In the future, we need to have a better understanding of the biological mechanisms surrounding COVID-19 to provide more specific information on the prognosis for specific cardiovascular diseases and more focused treatment (and possible prevention) of cardiovascular diseases in patients recovering from COVID-19.

In the battle against the COVID-19 pandemic, the management of patients with concurrent cardiovascular (CV) comorbidities poses significant challenges. As the Capone V, et al. pointed out (45), it is essential to incorporate more insights from the study by Capone V, et al. (45) Pharmacologically, anticoagulant use demands great caution. For COVID-19 patients with a history of atrial fibrillation, the infection's acute phase can disrupt the normal blood coagulation state, heightening the risk of bleeding according to Capone V et al. (45), clinicians must carefully weigh the pros and cons and precisely adjust the dosage of anticoagulants to prevent thrombosis while averting bleeding incidents. When antiviral drugs are co-administered with medications commonly used for cardiovascular conditions, extra vigilance is needed. Drugs like ACE inhibitors and ARBs for hypertension can interact with COVID-19 therapies. Doctors should closely monitor and adjust the drug type and dosage in a timely manner to maintain stable cardiac function. Non-pharmacological measures are equally crucial. Strengthened cardiovascular monitoring is a must. Regular electrocardiogram (ECG) checks can quickly detect arrhythmias; echocardiograms help to observe potential myocardial damage; tracking biomarkers such as troponin and BNP enables accurate assessment of the disease progression and prompts appropriate escalation of care plans.

Several potential study limitations need to be considered. First, most meta-analyses are highly heterogeneous, almost certainly due to measurement problems between studies and different samples, recruitment strategies, and follow-up times. Therefore, we used a random effects method in this meta-analysis to consider unmeasured inter-study factors. Second, our findings are limited by the lack of data on many symptoms, especially a combination of symptoms. The definition and reporting of symptoms are different in different studies, and although we have classified the symptoms as similar symptoms, this may introduce bias. Third, almost all studies came from high-income countries, which limits their universal applicability to low-and middle-income countries. Fourth, some studies used contemporary controls and did not rule out the possibility that some individuals may be infected with SARS-CoV-2, but were not yet tested. If such individuals exist in many of these contemporary controls, the results may be biased to the zero hypotheses. Fifth, due to the lack of non-hospitalization, hospitalization, intensive care and other patient data, our research is limited to subgroup analysis or meta regression analysis. Sixth, the included studies differed in defining Long COVID and one limitation is that included papers predate mass vaccination, preventing analysis of vaccination status's impact on long-term cardiovascular conditions (46–49). And we also cannot ascertain whether the COVID-19 group is healthier than the control group, which could potentially result in a spurious correlation between COVID and cardiovascular outcomes (45).

## Conclusion

This meta-analysis showed that the cardiovascular risk burden of long-term COVID-19 is significant and spans multiple categories of cardiovascular disease (ischemic and non-ischemic heart disease, arrhythmias, etc.). Care for survivors of COVID-19 after acute attack should include attention to cardiovascular health and disease.

## Data Availability

The original contributions presented in the study are included in the article/Supplementary Material, further inquiries can be directed to the corresponding authors.
