# Supplementary material for: Cardiovascular outcomes in long COVID-19: a systematic review and meta-analysis
Source: Front Cardiovasc Med. 2025 Jan 29;12:1450470. doi: 10.3389/fcvm.2025.1450470 (PMC11814196; doi:10.3389/fcvm.2025.1450470)

Long-term cardiovascular outcomes of COVID-19 in adults: a systematic review and meta-analysis

Supplementary files

Search strategy

Database(s): PubMed

Search Strategy:

#	Searches	Results
1	((((((((((((((COVID 19[Title/Abstract]) OR (SARS-CoV-2 Infection[Title/Abstract])) OR (Infection, SARS-CoV-2[Title/Abstract])) OR (SARS CoV 2 Infection[Title/Abstract])) OR (SARS-CoV-2 Infections[Title/Abstract])) OR (2019 Novel Coronavirus Disease[Title/Abstract])) OR (2019 Novel Coronavirus Infection[Title/Abstract])) OR (2019-nCoV Disease[Title/Abstract])) OR (Disease, 2019-nCoV[Title/Abstract])) OR (COVID 19 Virus Infection[Title/Abstract])) OR (Coronavirus Disease 19[Title/Abstract])) OR (COVID-19 Virus Disease[Title/Abstract])) OR (2019-nCoV Infection[Title/Abstract])) OR (COVID-19 Pandemic[Title/Abstract])) OR (COVID-19 Pandemics[Title/Abstract])	263894
2	(((((((((long-COVID[Title/Abstract]) OR (long-haul COVID[Title/Abstract])) OR (post-acute COVID syndrome[Title/Abstract])) OR (post-acute COVID19 syndrome[Title/Abstract])) OR (persistent COVID-19[Title/Abstract])) OR (long COVID[Title/Abstract])) OR (long hauler COVID[Title/Abstract])) OR (post-acute sequelae of SARS-CoV-2 infection[Title/Abstract])) OR (long haul COVID[Title/Abstract])) OR (chronic COVID syndrome[Title/Abstract])	2020
3	1 or 2	264203
4	((((((((associated disease) OR (sequelae[Title/Abstract])) OR (sequels[Title/Abstract])) OR (coexistent disease[Title/Abstract])) OR (concomitant disease[Title/Abstract])) OR (associated conditions[Title/Abstract])) OR (coexistent conditions[Title/Abstract])) OR (concomitant conditions[Title/Abstract])) OR (persistent symptom[Title/Abstract])	4457591
5	((((((((Cohort Study[Title/Abstract]) OR (Cohort Study Studies, Cohort[Title/Abstract])) OR (Study, Cohort[Title/Abstract])) OR (Analyses, Cohort[Title/Abstract])) OR (Historical Cohort Studies[Title/Abstract])) OR (Cohort Study, Historical[Title/Abstract])) OR (Historical Cohort Study[Title/Abstract])) OR (Studies, Historical Cohort[Title/Abstract])) OR (Incidence Studies[Title/Abstract])	275344
6	#3 AND #4 AND #5	3802

Database(s): Embase

Search Strategy:

#	Searches	Results
1	'COVID 19':ab,ti OR 'SARS-CoV-2 Infection':ab,ti OR 'Infection, SARS-CoV-2':ab,ti OR 'SARS CoV 2 Infection':ab,ti OR 'SARS-CoV-2 Infections':ab,ti OR '2019 Novel Coronavirus Disease':ab,ti OR '2019 Novel Coronavirus Infection':ab,ti OR '2019-nCoV Disease':ab,ti OR '2019 nCoV Disease':ab,ti OR '2019-nCoV Diseases':ab,ti OR 'Disease, 2019-nCoV':ab,ti OR 'COVID-19 Virus Infection':ab,ti OR 'COVID 19 Virus Infection':ab,ti OR 'COVID-19 Virus Infections':ab,ti OR 'Infection, COVID-19 Virus':ab,ti OR 'Virus Infection, COVID-19':ab,ti OR 'Coronavirus Disease 2019':ab,ti OR 'Disease 2019, Coronavirus':ab,ti OR 'Coronavirus Disease-19':ab,ti OR 'Coronavirus Disease 19':ab,ti OR 'Severe Acute Respiratory Syndrome Coronavirus 2 Infection':ab,ti OR 'SARS Coronavirus 2 Infection':ab,ti OR 'COVID-19 Virus Disease':ab,ti OR 'COVID 19 Virus Disease':ab,ti OR 'COVID-19 Virus Diseases':ab,ti OR 'Disease, COVID-19 Virus':ab,ti OR 'Virus Disease, COVID-19':ab,ti OR '2019-nCoV Infection':ab,ti OR '2019 nCoV Infection':ab,ti OR '2019-nCoV Infections':ab,ti OR 'Infection, 2019-nCoV':ab,ti OR 'COVID19':ab,ti OR 'COVID-19 Pandemic':ab,ti OR 'COVID 19 Pandemic':ab,ti OR 'Pandemic, COVID-19':ab,ti OR 'COVID-19 Pandemics'	282772
2	'long-COVID':ab,ti OR 'long-haul COVID':ab,ti OR 'post-acute COVID syndrome':ab,ti OR 'persistent COVID-19':ab,ti OR 'post-acute COVID19 syndrome':ab,ti OR 'long hauler COVID':ab,ti OR 'long COVID':ab,ti OR 'post-acute sequelae of SARS-CoV-2 infection':ab,ti OR 'long haul COVID':ab,ti OR 'chronic COVID syndrome'	2009
3	1 or 2	283175
4	'associated disease':ab,ti OR 'sequelae':ab,ti OR 'sequels':ab,ti OR 'coexistent disease':ab,ti OR 'concomitant disease':ab,ti OR 'associated conditions':ab,ti OR 'coexistent conditions':ab,ti OR 'concomitant conditions':ab,ti OR 'persistent symptom'	111912
5	'Cohort Study':ab,ti OR 'Studies, Cohort':ab,ti OR 'Study, Cohort':ab,ti OR 'Analysis, Cohort':ab,ti OR 'Analyses, Cohort':ab,ti OR 'Cohort Analyses':ab,ti OR 'Cohort Analysis':ab,ti OR 'Historical Cohort Studies':ab,ti OR 'Cohort Studies, Historical':ab,ti OR 'Cohort Study, Historical':ab,ti OR 'Historical Cohort Study':ab,ti OR 'Study, Historical Cohort':ab,ti OR 'Studies, Historical Cohort':ab,ti OR 'Incidence Studies':ab,ti OR 'Incidence Study':ab,ti OR 'Studies, Incidence':ab,ti OR 'Study, Incidence'	418276

6	#3 AND #4 AND #5	266

Web of Science

Search strategy:

#	Searches	Results
1	TS = (COVID 19 OR SARS-CoV-2 Infection OR Infection, SARS-CoV-2 OR SARS CoV 2 Infection OR SARS-CoV-2 Infections OR 2019 Novel Coronavirus Disease OR 2019 Novel Coronavirus Infection OR 2019-nCoV Disease OR 2019 nCoV Disease OR 2019-nCoV Diseases OR Disease, 2019-nCoV OR COVID-19 Virus Infection OR COVID 19 Virus Infection OR COVID-19 Virus Infections OR Infection, COVID-19 Virus OR Virus Infection, COVID-19 OR Coronavirus Disease 2019 OR Disease 2019, Coronavirus OR Coronavirus Disease-19 OR Coronavirus Disease 19 OR Severe Acute Respiratory Syndrome Coronavirus 2 Infection OR SARS Coronavirus 2 Infection OR COVID-19 Virus Disease OR COVID 19 Virus Disease OR COVID-19 Virus Diseases OR Disease, COVID-19 Virus OR Virus Disease, COVID-19 OR 2019-nCoV Infection OR 2019 nCoV Infection OR 2019-nCoV Infections OR Infection, 2019-nCoV OR COVID19 OR COVID-19 Pandemic OR COVID 19 Pandemic OR Pandemic, COVID-19 OR COVID-19 Pandemics)	286244
2	TS=(long-COVID OR long-haul COVID OR post-acute COVID syndrome OR persistent COVID-19 OR post-acute COVID19 syndrome OR long hauler COVID OR long COVID OR post-acute sequelae of SARS-CoV-2 infection OR long haul COVID OR chronic COVID syndrome)	28372
3	1 or 2	286456
4	AB=(associated disease OR sequelae OR sequels OR coexistent disease OR concomitant disease OR associated conditions OR coexistent conditions OR concomitant conditions OR persistent symptom)	3515752
5	AB=(Cohort Study OR Studies, Cohort OR Study, Cohort OR Analysis, Cohort OR Analyses, Cohort OR Cohort Analyses OR Cohort Analysis OR Historical Cohort Studies OR Cohort Studies, Historical OR Cohort Study, Historical OR Historical Cohort Study OR Study, Historical Cohort OR Studies, Historical Cohort OR Incidence Studies OR Incidence Study OR Studies, Incidence OR Study, Incidence)	1632353

6	#3 AND #4 AND #5	1411

Cochrane COVID-19 Study Register

Results: 850

A series of separate searches were run. Results were added to RIS File. Only studies that included results were added.

Search 1: 70

longcovid* or "long covid*" or longcoronavirus* or longcoronavirinae or "long coronavirinae" or longCov or "long Cov" or longsars or "long sars"

Search 2: 256

ongoing symptom or persistent symptom

Search 3: 44

Sequelae

Search 4: 480

postcovid* or "post covid*" or postcoronavirus* or postcoronavirinae or "post coronavirinae" or postCov or "post Cov" or postsars or "post sars"

ProQuest Coronavirus Research Database

Number of results: 132

noft(("long covid" OR longcovid OR longcoronavirus OR "long coronavirus" OR "long COVID*" OR (persist* AND covid) OR "post acute covid" OR "post covid" OR "post-covid" OR "post infection" OR postviral* OR "post viral*" OR postvirus* OR "post virus*" OR "sequela* covid" OR (post discharg* AND covid) "long sars*" OR "post-acute SARS-CoV-2" OR "Late sequelae covid*" OR "post acute COVID*" OR "Covid* syndrome" OR "post-acute sequelae SARS-CoV-2 infection" OR "long haul*" OR long-haul* OR longcovid OR "long coronavirus" OR "long COVID-19" OR (persist* AND covid) OR "post acute covid" OR "post-covid"))

COVID-19 Living Overview of the Evidence (L-OVE) subset of Episteminokos database:

Search results: 45

("long covid" OR "post acute covid" OR "post covid" OR "post-covid" OR longcovid OR longcoronavirus OR "long coronavirus" OR "long covid syndrome" or "post-acute sequelae SARS-CoV-2 infection")

WHO COVID-19 Global literature on coronavirus disease

Number of results: 134

(tw:(("long covid" OR longcovid OR longcoronavirus OR "long coronavirus" OR "long COVID*" OR (persist* AND covid) OR "post acute covid" OR "post covid" OR "post-covid" OR "post infection" OR postviral* OR "post viral*" OR postvirus* OR "post virus*" OR “sequela* covid” OR (post discharg* AND covid) "long sars*" OR "post-acute SARS-CoV-2" OR "Late sequelae covid*" OR "post acute COVID*" or "Covid* syndrome" or "post-acute sequelae SARS-CoV-2

infection" or "long haul*" or long-haul* or longcovid or "long coronavirus" or "long COVID-19" or (persist* and covid) or "post acute covid" or "post-covid")))

Table S1: Risk of bias in Cohort Studies

NEWCASTLE-OTTAWA QUALITY ASSESSMENT SCALE COHORT STUDIES

Study	Selection	Comparability	Outcome	AHRQ standard	Total # of stars
Xie, 2022	★★★	★★★	★★★	High	9
Potts,2022	★★★	★★★	★★★	High	9
Subramanian, 2022	★★★	★★★	★★★	High	9
Walker, 2021	★★★	★★★	★★★	High	9
Cohen, 2022	★★★	★★★	★★★	High	9
Rivera, 2022	★★	★★	★★★	High	7
Ayoubkhani, 2021	★★★	★★★	★★★	High	9
Dautherty, 2021	★★★	★★★	★★★	High	9

Note: **NA**= Not Applicable, **Red**=poor/low, **orange**=fair/moderate, **green**=good/high, **AHRQ**= Agency for Healthcare Research and Quality Thresholds for converting the Newcastle-Ottawa scales to AHRQ standards (good, fair, and poor): **Good/high quality:** 3 or 4 stars in selection domain AND 1 or 2 stars in comparability domain AND 2 or 3 stars in outcome/exposure domain **fair/moderate quality:** 2 stars in selection domain AND 1 or 2 stars in comparability domain AND 2 or 3 stars in outcome/exposure domain | **poor/low quality:** 0 or 1 star in selection domain OR 0 stars in comparability domain OR 0 or 1 stars in outcome/exposure domain

- Figure S1. SARS-CoV-2 group versus comparison group for risk difference per 100 individuals (A)and hazard ratio (B) for **coronary disease** in long COVID-19.

A:

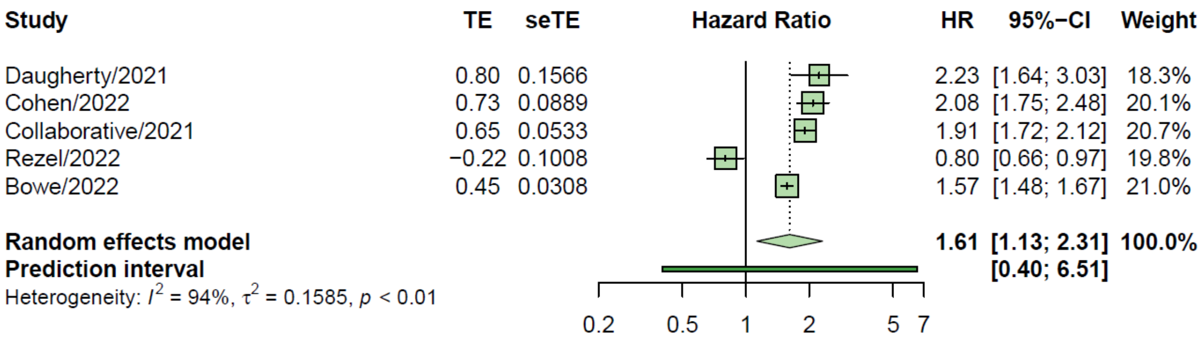

B:

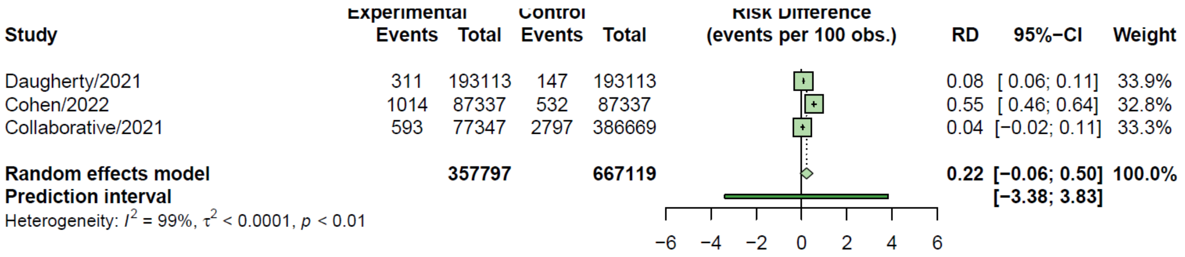

- Figure S2. SARS-CoV-2 group versus comparison group for risk difference per 100 individuals (A)and hazard ratio (B) for **Stroke** in long COVID-19.

A:

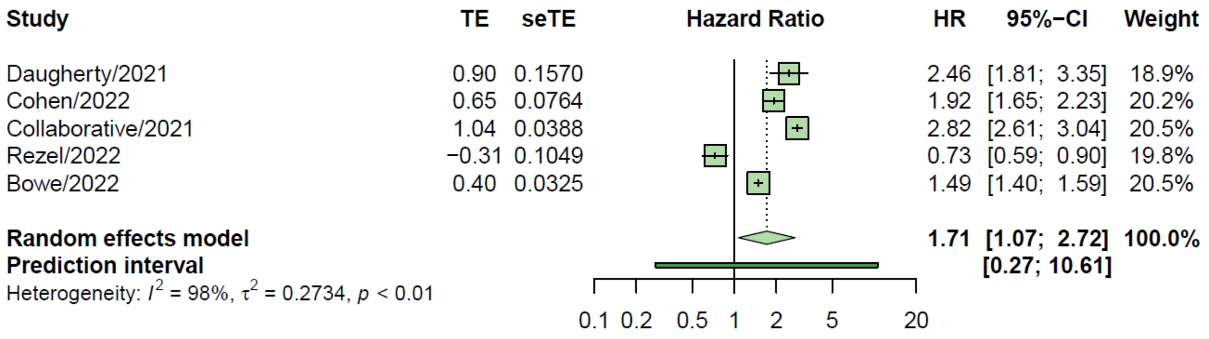

B:

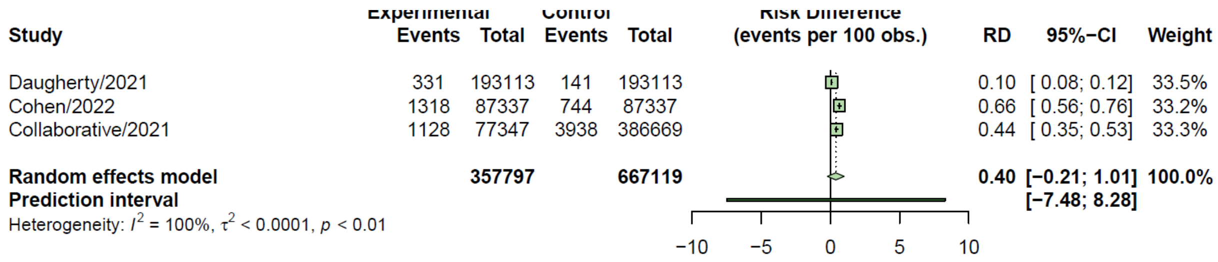

- Figure S3. SARS-CoV-2 group versus comparison group for risk difference per 100 individuals (A)and hazard ratio (B) for **arrhythmia** in long COVID-19.

A:

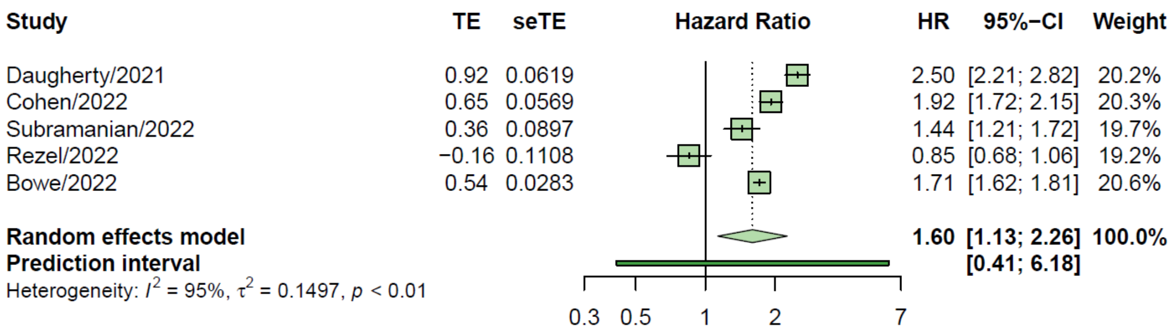

B:

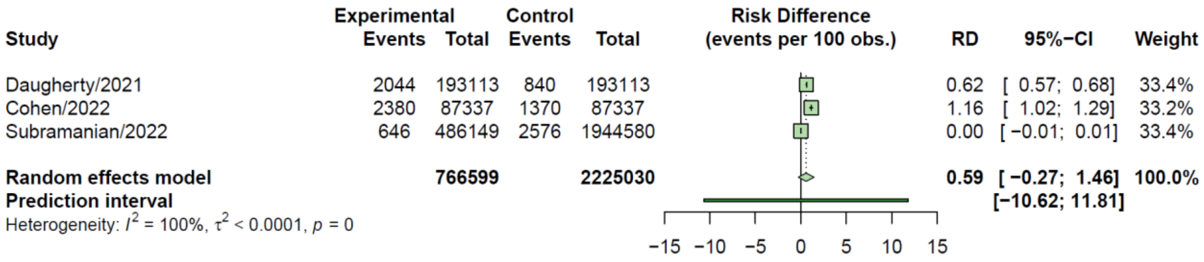

- Figure S4. SARS-CoV-2 group versus comparison group for risk difference per 100 individuals (A)and hazard ratio (B) for **cardiomyopathy and myocarditis** in long COVID-19.

A:

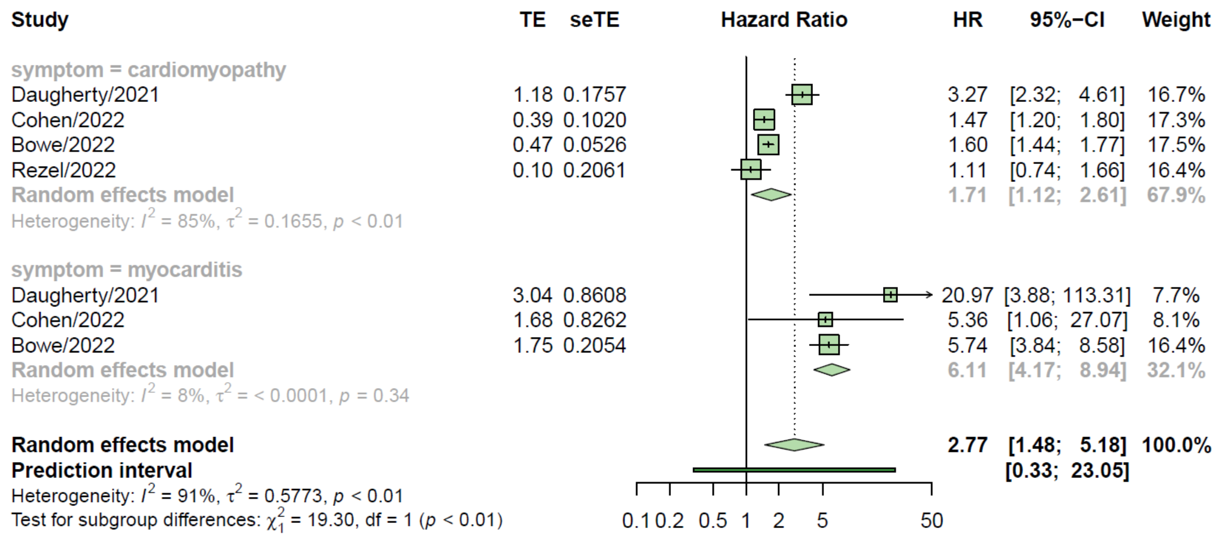

B:

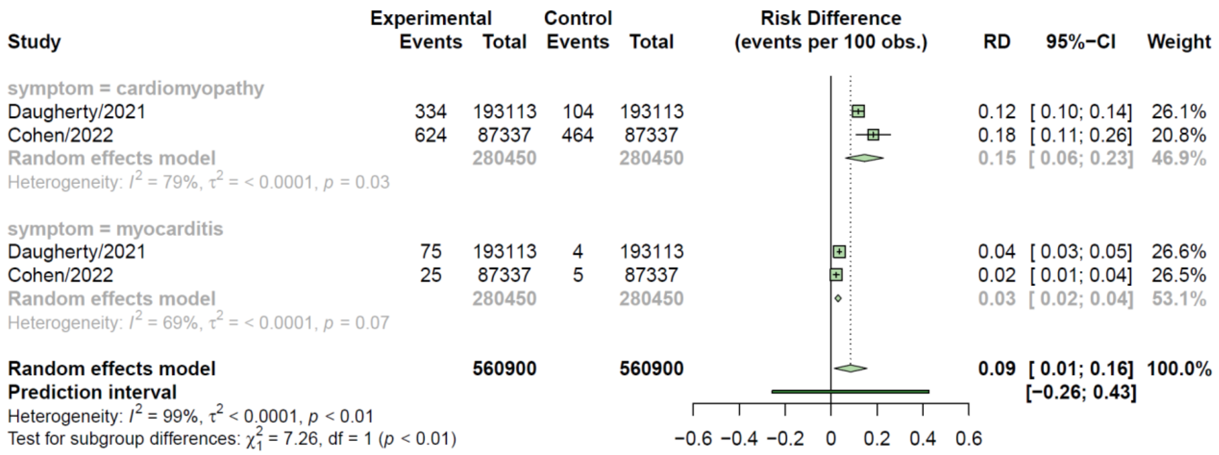

- Figure S5. SARS-CoV-2 group versus comparison group for risk difference per 100 individuals (A)and hazard ratio (B) for **hypertension** in long COVID-19.

A:

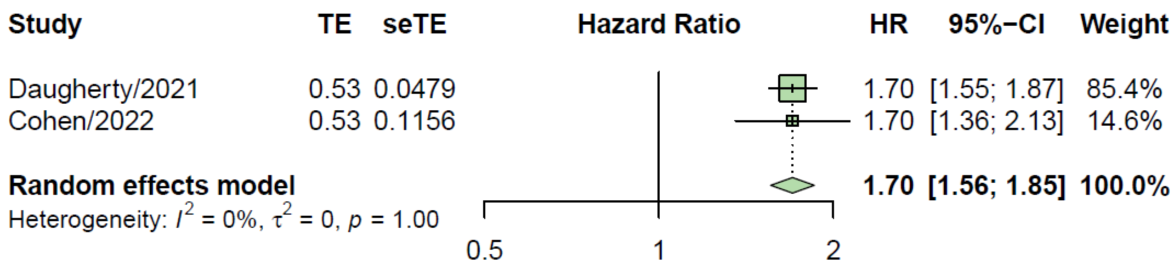

B:

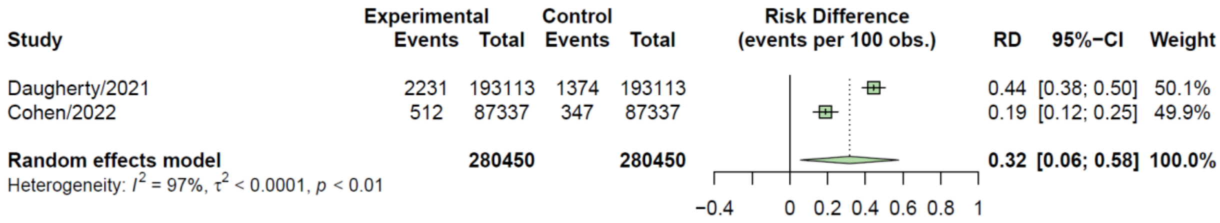

- Figure S6. SARS-CoV-2 group versus comparison group for risk difference per 100 individuals (A)and hazard ratio (B) for **heart failure** in long COVID-19.

A:

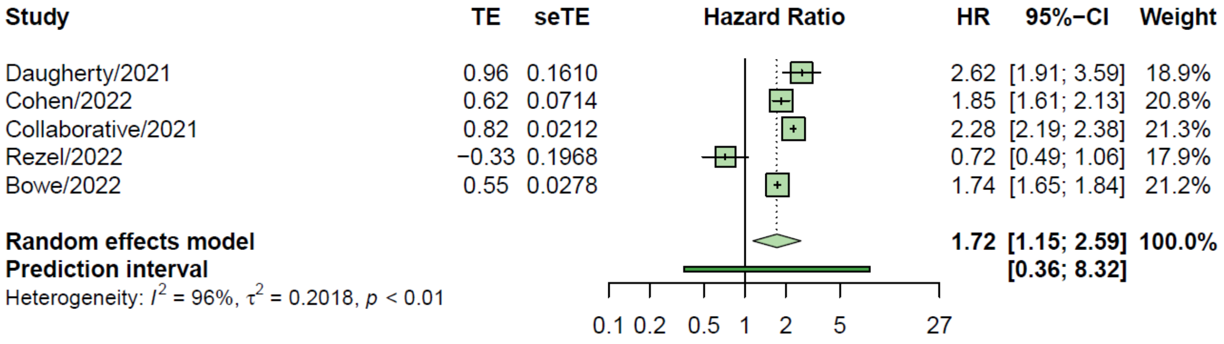

B:

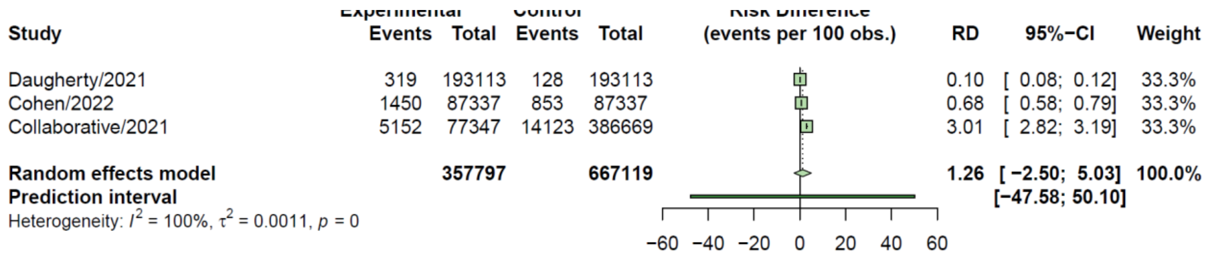

- Figure S7. SARS-CoV-2 group versus comparison group for risk difference per 100 individuals (A)and hazard ratio (B) for **cardiogenic shock** in long COVID-19.

A:

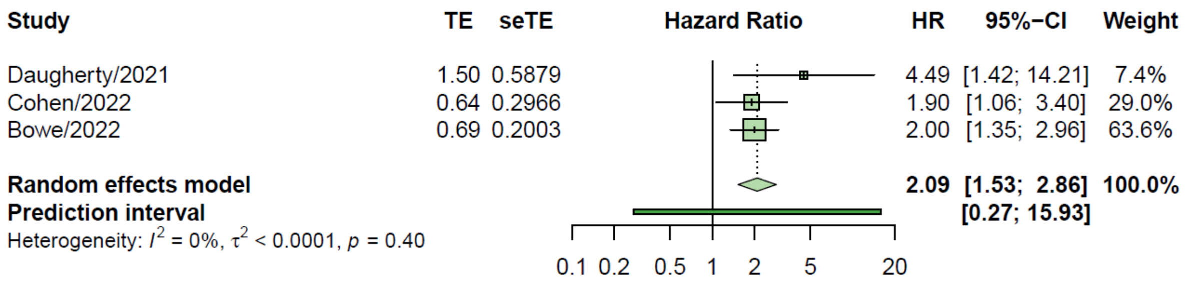

B:

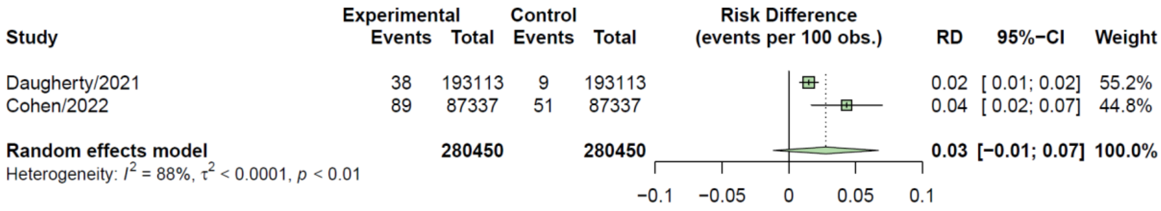

Supplement: Supplementary file 2 [file Datasheet1.pdf]
